# Urinary bacterial profile and antibiotic susceptibility pattern among pregnant women in Rahima Moosa Mother and Child Hospital, Johannesburg

**DOI:** 10.4102/sajid.v37i1.343

**Published:** 2022-01-28

**Authors:** Ogbonnaya Orji, Zandile Dlamini, Amy J. Wise

**Affiliations:** 1 Department of Obstetrics and Gynaecology, Faculty of Health Sciences, University of the Witwatersrand, Johannesburg, South Africa

**Keywords:** UTI, sensitivity, urine microscopy, sensitivity and culture, pathogen, pregnancy

## Abstract

**Background:**

Urinary tract infection (UTI) in pregnancy is associated with significant morbidity for both the mother and the foetus. The aim of this study was to determine the prevalence of UTI, urinary bacterial susceptibility, and resistance patterns among pregnant women with a possible UTI at Rahima Moosa Mother and Child Hospital (RMMCH) in Johannesburg.

**Methods:**

In this retrospective study, we analysed mid-stream urine culture and antibiotic susceptibility data from both inpatients and outpatients of pregnant women who attended RMMCH from January 2017 to December 2017. Data were collected from patients’ files and then matched with urine microscopy, sensitivity and culture (MC&S) results from the National Health Laboratory Services (NHLS) data.

**Results:**

Urine microscopy, cultures and sensitivities were performed on 1984 specimens belonging to pregnant women who presented with symptoms and/or signs of a UTI. A total of 333 patients (16.8%) had positive bacterial cultures. *Escherichia coli* (E. coli) was the commonest bacterial isolate (49.9%). Other microorganisms isolated included Klebsiella species (14.4%), Enterococcus faecalis (12.9%) and coagulase-negative staphylococci (CoNS); (8.9%). Approximately 98% of organisms were sensitive to cephalexin. Cefuroxime (95.2%), ceftriaxone/cefotaxime (94.4%) and nitrofurantoin (81.9%) demonstrated antimicrobial effectiveness as indicated. Most isolates were resistant to ampicillin/amoxicillin (84.4%), Trimethoprim/Sulfamethoxazole (55.6%) and amoxicillin-clavulanic acid (50.2%).

**Conclusion:**

*E. coli* was the commonest pathogen causing UTIs in pregnancy with Enterococcus faecalis increasing in prevalence. The choice of antimicrobial therapy in pregnancy should be determined according to sensitivity and resistance and foeto-maternal safety.

## Introduction

Several changes occur during pregnancy that predispose pregnant women to urinary tract infections (UTIs). Physiological, physical, mechanical and hormonal changes result in increased urinary stasis. Altered urine composition with elevated glucose levels coupled with a short urethra (3 cm – 4 cm in women) increases the predisposition to UTIs in pregnant women.^1^ The prevalence of UTI in pregnancy ranges between 2% and 10% globally.^2,6,7,8^

Urinary tract infections are among the commonest bacterial infections complicating pregnancy.^2,3,4^ Urinary tract infection can be either symptomatic or asymptomatic. A symptomatic UTI patient is one with significant bacteriuria and with symptoms of a UTI. Whereas, a condition characterised by lack of symptoms of UTI with significant bacterial yielding positive urine cultures (≥ 10^5^ colony forming units/millilitre [CFU/mL]) is called an asymptomatic UTI (asymptomatic bacteriuria).^5^

However, symptoms like dysuria and frequency are common in pregnancy, but have a very low specificity for a true UTI. Other symptoms such as urethritis, cystitis or pyelonephritis may be present. Clinicians can be wrong in their suspicion of a UTI from the symptoms alone. Many of those with symptoms and positive cultures may therefore have had asymptomatic bacteriuria instead.^6^ The significance of UTI in pregnancy, in view of its associated maternal and foetal morbidity and mortality, has been widely evaluated.

The occurrence of UTI in pregnancy is increased by several factors. The highest incidence has been reported in African-American multiparous women, while the lowest incidence occurs among affluent white women of low parity.^6^ Poor socio-economic status is a significant risk factor, with indigent women having a five-fold increased risk of acquiring UTIs.^9^ Other risk factors include: increasing maternal age, high parity, reduced immune function, poor perineal hygiene, a history of recurrent UTI, diabetes mellitus, neurogenic urinary retention, anatomic or functional urinary tract abnormalities, and increased frequency of sexual activity.^2,10,11^

Studies in developing countries show that UTIs are usually present at the first antenatal visit and less than 1% of women develop bacteriuria after a negative screen in early pregnancy.^2^ A UTI in pregnancy contributes to significant maternal and perinatal morbidity and mortality. Maternal complications include overt pyelonephritis in 25% – 40% of previously asymptomatic women as the pregnancy progresses, and in 1% – 2% in those with symptomatic infections.^2,12^ Other maternal complications include: anaemia, miscarriages, preterm labour, hypertension, pre-eclampsia, puerperal sepsis, chronic pyelonephritis and occasionally, renal failure.^2,8,13^ Urinary tract infections are also associated with foetal growth restriction, prematurity, low birthweight and foetal death.^13,14^

The causative organisms arise from the normal vaginal, perineal and faecal flora.^3,13^ These include: *Escherichia coli (E. coli), Staphylococcus aureus, Enterococcus faecalis, Proteus mirabilis, Klebsiella species, and Streptococcus species,* among others.^2^ There are numerous reports of resistance to antimicrobials by urinary tract pathogens.^14,15^ Antimicrobial resistance in these organisms occurs because of broad-spectrum antibiotic abuse in humans and in animal feeds.^13,14^

Antibiotic resistance is frequently observed in nosocomial settings. However, it is also becoming apparent in community-acquired UTIs, with an increasing incidence of Gram-positive cocci, for example, Staphylococci sp. and Gram-negative organisms such as Klebsiella sp. becoming more prevalent.^2,16,17^

Urinary tract infections may present as acute infections and the administration of antibiotics may be necessary while awaiting microscopy, sensitivity and culture (MC&S) results to prevent and/or reduce maternal and foetal morbidity and mortality especially in low-resourced countries.

The aim of this study was to determine the antibiotic sensitivity pattern among pregnant women with symptomatic UTIs and to describe the pathogenicity and antibiotic susceptibility among the causative bacterial organisms.^18^

Knowledge of the local bacterial and susceptibility patterns can guide the judicious use of empiric therapy.^19^

## Methods

### Setting and study design

The study was conducted at the Rahima Moosa Mother and Child Hospital (RMMCH), which is a regional hospital. The hospital serves a population of approximately 200 000 women and children in three regions in the Gauteng province.^20^ An average of 1700 women attend the antenatal clinic on a monthly basis, including high- and low-risk women.

In this retrospective study, we analysed mid-stream urine culture and antibiotic susceptibility data from both inpatients and outpatients of pregnant women who attended RMMCH from January 2017 to December 2017. The National Health Laboratory Services (NHLS) was approached for a list of all samples sent in the specified time period. All available files from those that were culture positive were retrieved and reviewed. The NHLS data uses a pre-defined procedure for culturing, bacterial identification, and susceptibility testing.

### Urine sample collection, primary inoculation, and analysis

The samples were collected during routine clinical care, at the discretion of the treating clinician, and it is not possible to comment on the correctness of the technique or time taken to reach the laboratory. The reason for sending a sample was not always reflected in the available notes.

Urine samples were cultured on 5% blood agar and MacConkey agar using calibrated loops in a semi-quantitative assessment and incubated in aerobic conditions at 35 °C – 37 °C for 18 h – 24 h.^2^ Isolates were identified and confirmed using standard methods including Gram staining; colony morphology on media; growth on selective media; lactose and mannitol fermentation; hydrogen sulphide production; catalase, oxidase, coagulase, and indole tests; citrate utilisation; and urease testing. Urine infection cultures were considered positive with bacterial counts ≥ 10^5^/mL.^2,21^ All patients with positive urine cultures were treated.^2^

### Antimicrobial susceptibility testing

For reliable detection, laboratories may use conventional, quantitative susceptibility testing methods or specially developed, single concentration agar screening tests for some resistant species.^2^

Antimicrobial susceptibility testing for UTIs in the laboratory is performed using two groups of antimicrobial discs for cascade reporting, a strategy recommended by the Clinical and Laboratory Standards Institute. In this strategy, the reporting of antimicrobial susceptibility test results for the second group of agents (e.g. broader- spectrum, more costly) may only be reported if an organism is resistant to primary agents within a particular drug class. If a pathogen shows resistance to all of these, the laboratory will move to the second stage for testing broad-spectrum antibiotics.^2^

Identification and sensitivity testing were done if the culture was pure and growth was significant (≥ 10^5^ CFU/mL). If the culture growth involved a mix of two pathogens and no isolate was dominant, or more than two types of colonies were grown, then it was reported as a mixed growth (contaminants) and clinical correlation was needed to make a determination. In such cases, no sensitivity testing was carried out.^2,22^

### Statistical analysis

All the data collected were managed using Research Electronic Data Capture (REDCap) electronic data capture tools hosted at a University in Johannesburg. The REDCap is a secure, web-based application designed to support data capture for research studies.^23,24^ The biostatisticians at the University of the Witwatersrand (WITS) in Johannesburg assisted in the analysis phase of the study. The data were analysed using Stata® version 13.0.15.^25^ Descriptive data was expressed using means with ranges and medians with standard deviations (s.d.).

### Ethical considerations

Ethical clearance was obtained from the Human Research Ethics Committee (Medical) of the University of the Witwatersrand, reference number: M181070.

## Results

**FIGURE 1:**
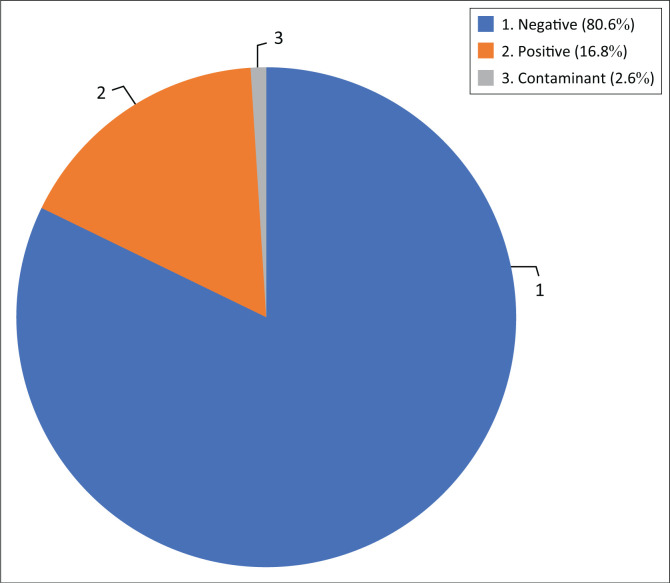
Culture results of urine specimens submitted for microscopy, sensitivity and culture.

A total of 1984 urine samples from pregnant women with suspected UTI were selected for isolation and identification of bacteria and antimicrobial susceptibility testing in both inpatients and outpatients at our centre, 333 urine samples (16.8%) were culture positive for UTI, while 1599 urine samples (80.6%) were culture negative and 52 urine samples (2.6%) were identified as contaminants and contaminated urine were determined by more than five epithelial cells on microscopic evaluation, specific gravidity > 1.035 and urine culture with polymicrobial growth – that is, urine culture that contain more than one organisms (Figure 1).

In all, 324 women (97.3%) were booked for antenatal care. The ages of the study cohort ranged between 16 and 44 years, with the majority (58.9%) in the 20 and 29-year age group. There were 52 (15.6%) human immunodeficiency virus (HIV) infected women. The highest frequency of UTIs observed (*n* = 229, 68.8%) were from specimens taken in the third trimester. Gestational ages ranged between 26 weeks and 40 weeks and birthweights from 619 g to 4495 g. Of the 333 patients, 29.1% (97/333) were inpatients and 70.9% (236/333) were outpatients (Table 1).

**TABLE 1:** Demographic, antenatal and treatment data of women with positive microscopy, sensitivity and cultures.

Variable	Number *N* = 333	Percentages (%)
**Age (years)**		
< 20	39	11.7
20–29	170	51.1
30–39	110	33.0
> 40	14	4.2
**Parity**		
0	132	39.7
1	68	20.4
2	81	24.3
3	36	10.8
4	15	4.5
5	1	0.3
**Gravidity**		
1	106	31.9
2	72	21.6
3	75	22.5
4	45	13.5
5	18	5.4
6	16	4.8
7	1	0.3
**Trimester during which urine MC&S was done**		
First trimester	2	0.6
Second trimester	102	30.6
Third trimester	229	68.8
**ANC attendance (at least once)**		
Booked	324	97.3
Unbooked	9	2.7
**HIV**		
Negative	281	84.4
Positive	52	15.6
**Recurrent infection**	5	1.50
**Admission status**		
Inpatient	97	29.1
Outpatient	236	70.9

MC&S, microscopy, sensitivity and culture; ANC, antenatal care; HIV, human immunodeficiency virus.

The most frequently identified microorganism was *E. coli* (49.9%), followed by *Klebsiella* (14.4%), *Enterococci faecalis* (12.9%) and coagulase-negative *Staphylococci* (CoNS) (8.9%) (Table 2).

**TABLE 2:** Number and percentage of microorganisms isolated from pregnant women urine samples in the study.

Microorganism	Number (*N*)	Percentage (%)
*Escherichia coli*	166	49.9
*Klebsiella species*	48	14.4
*Enterococcus faecalis* (Group D streptococci)	43	12.9
Coagulase- negative *staphylococci* (CoNS)	30	8.9
*Proteus*	3	0.9
*Enterobacter species*	3	0.9
*Streptococcus agalactiae* (Group B)	15	4.5
*Staphylococcus* spp, (other non-CoNS or S. aureus)	8	2.4
*Staphylococcus aureus*	8	2.4
*Acinetobacter*	2	0.7
Other Microorganisms (*Corynebacterium, Pseudomonas*)	7	2.1

CoNS, coagulase-negative staphylococci.

Table 3 shows that microbes demonstrated high susceptibility to cephalosporin: cephalexin (98.0%), cefuroxime (95.1%) and ceftriaxone/cefotaxime (94.4%), while 81.9% of the isolates were sensitive to nitrofurantoin. Piperacillin/tazobactam and gentamicin had an overall susceptibility of 96.2% and 70.1% respectively. The resistance observed in this study was gentamicin (29.7%), nitrofurantoin (13.5%) and piperacillin/tazobactam (2.0%), while intermediate resistance was 0.2% for gentamicin, 1.8% for piperacillin/tazobactam and 4.6% for nitrofurantoin.

**TABLE 3:** Overall sensitivity of antimicrobial agents.

Antimicrobial agents	Sensitive		Resistant		Intermediate		Total
	*N*	%	*N*	%	*N*	%	
Amikacin†	198	100	-	-	-	-	198
Ampicillin/Amoxicillin	48	15.0	273	84.4	2	0.6	324
Amoxicillin + clavulanic acid	146	47.3	155	50.2	8	2.5	309
Cefotaxime/Ceftriaxone	291	94.4	17	5.6	-	-	308
Ceftazidime	260	92.0	22	8.0	-	-	282
Cefuroxime	314	95.2	16	4.8	-	-	330
Cephalexin/Cephradine	323	98	7	2	-	-	330
Chloramphenicol	6	100	-	-	-	-	6
Ciprofloxacin	180	80.2	41	18.4	3	1.4	224
Clindamycin	10	40.0	13	55.5	1	5.5	24
Colistin Sulphate	2	100	-	-	-	-	2
Ertapenem†	118	100	-	-	-	-	118
Erythromycin/Azithromycin	8	16.7	34	70.8	6	12.5	48
Gentamicin	211	70.1	90	29.7	1	0.2	302
Imipenem†	203	100	-	-	-	-	203
Linezolid	101	100	-	-	-	-	101
Meropenem†	107	98.2	2	1.8	-	-	109
Moxifloxacin	48	88.0	2	4.0	4	8.0	54
Nalidixic acid	13	18.9	55	81.1	-	-	68
Nitrofurantoin	271	81.9	45	13.5	15	4.6	331
Piperacillin/Tazobactam	92	96.2	2	2.0	2	1.8	96
Tigecycline†	69	100	-	-	-	-	69
Tobramycin	69	84.2	8	9.3	5	6.5	82
Trimethoprim/Sulfamethoxazole	129	44.4	161	55.6	-	-	290
Vancomycin	78	100	-	-	-	-	78

*N,* number, %, percentages.

† , Provided as single discs.

Amikacin, chloramphenicol, imipenem, linezolid, tobramycin, ertapenem, vancomycin and colistin sulphate had the highest overall potency of 100% each to all isolated microbials tested against them. Co-amoxiclav, cotrimoxazole, ampicillin/amoxicillin, clindamycin, nalidixic acid and erythromycin/azithromycin were less than 50% effective against the cultured microbes.

Table 4 shows antimicrobial sensitivity to gram-negative microbials. Regarding *E. coli*, most cultures were 100% sensitive to amikacin, imipenem, ertapenem, meropenem. However, the majority were resistant to nalidixic acid, ampicillin/amoxicillin and co-amoxiclav.

**TABLE 4:** Antimicrobial sensitivity in gram-negative bacterial isolates.

Gram-negative bacteria/Antimicrobial	*Escherichia coli* (166)			*Klebsiella* (48)			*Enterobacter* (3)			*Proteus* (3)			*Acinetobacter* (2)		
	S	R	I	S	R	I	S	R	I	S	R	I	S	R	I
**Amikacin**															
*N*	166	-	-	48	-	-	3	-	-	3	-	-	-	-	-
%	100	-	-	100	-	-	100	-	-	100	-	-	-	-	-
**Ampicillin/Amoxicillin**															
*N*	59	104	3	-	43	-	-	-	-	2	-	-	-	2	-
%	35.7	62.8	1.4	-	100	-	-	-	-	100	-	-	-	100	-
**Co-amoxiclav**															
*N*	80	80	6	27	14	7	1	2	-	2	1	-	-	2	-
%	48	47.6	3.4	56.1	29.3	14.6	16.7	83.3	-	75	25	-	-	100	-
**Cephalexin/Cephradine**															
*N*	106	60	-	24	24	-	1	2	-	3	-	-	-	2	-
%	63.8	36.2	-	50	50	-	25	75	-	100	-	-	-	100	-
**Ceftriaxone**															
*N*	88	78	-	26	22	-	2	1	-	1	2	-	1	1	-
%	52.9	47.1	-	53.7	46.7	-	75	25	-	15	75	-	50	50	-
**Cefuroxime**															
*N*	100	64	2	24	24	-	-	-	-	3	-	-	-	2	-
%	60.5	39	0.5	50	50	-	-	-	-	100	-	-	-	100	-
**Ciprofloxacin**															
*N*	126	40	-	47	1	-	3	-	-	3	-	-	1	1	-
%	75.8	31.2	-	97.3	2.7	-	100	-	-	100	-	-	50	50	-
**Co-trimoxazole**															
*N*	55.1	43.9	-	28	20	-	3	-	-	83.3	16.7	-	1	1	-
%	-	-	-	58.1	41.9	-	100	-	-	-	-	-	50	50	-
**Ertapenem**															
*N*	166	-	-	28	-	-	3	-	-	3	-	-	2	-	-
%	100	-	-	100	-	-	100	-	-	100	-	-	100	-	-
**Gentamicin**															
*N*	140	25	1	34	14	-	2	1	-	3	-	-	1	1	-
%	84.4	15.0	0.6	70.1	28.9	-	83.3	16.7	-	100	-	-	50	50	-
**Imipenem**															
*N*	166	-	-	43	-	-	3	-	-	3	-	-	2	-	-
%	100	-	-	100	-	-	100	-	-	100	-	-	100	-	-
**Nalidixic acid**															
*N*	63	103	-	47	1	-	2	1	-	2	1	-	-	-	2
%	37.8	62.2	-	95	35	-	75	25	-	75	25	-	-	-	100
**Nitrofurantoin**															
*N*	126	40	-	10	22	16	1	2	-	-	3	-	-	-	2
%	75.5	25.5	-	20.9	44.9	34.2	25	75	-	-	100	-	-	-	100
**Tigecycline**															
*N*	100	66	-	36	12	-	3	-	-	3	-	-	NR	NR	NR
%	60.0	40.0	-	75	25	-	100	-	-	100	-	-	-	-	-
**Meropenem**															
*N*	166	-	-	43	-	-	2	-	-	2	-	-	1	1	-
%	100	-	-	100	-	-	100	-	-	100	-	-	50	50	-
**Piperacillin/Tazobactam**															
*N*	159	2	5	40	-	-	3	-	-	3	-	-	1	1	-
%	95.7	1.4	2.9	100	-	-	100	-	-	100	-	-	50	50	-
**Tobramycin**															
*N*	147	13	6	37	11	-	-	-	-	-	-	-	-	-	-
%	88.5	7.7	3.8	77.8	22.2	-	-	-	-	-	-	-	-	-	-
**Colistin sulphate**															
*N*	NR	NR	NR	NR	-	-	-	-	-	-	-	-	2	-	-
%	-	-	-	-	-	-	-	-	-	-	-	-	100	-	-

S, sensitive; R, resistant; I, intermediate; NR, not recorded; *N*, numbers; %, percentages.

All the *Klebsiella* were sensitive to amikacin, ertapenem and imipenem, while nearly half of *Klebsiella* isolates were sensitive to different cephalosporins whereas only 20.9% were sensitive to nitrofurantoin. *Enterobacter* species showed 100% sensitivity to amikacin, ciprofloxacin, co-trimoxazole, ertapenem and imipenem. These cultures were most resistant to co-amoxiclav and cefuroxime. The three *Proteus* species were sensitive to most tested antibiotics except ceftriaxone, with a 75% reported resistance.

*Acinetobacter* species was cultured twice and showed resistance to ampicillin/amoxicillin, co-amoxiclav, cefuroxime, nalidixic acid and nitrofurantoin. Both were sensitive to the carbapenems.

All *Enterococcus faecalis* culture were sensitive to ampicillin/amoxicillin, co-amoxiclav, nitrofurantoin and vancomycin, but were all resistant to co-trimoxazole, gentamicin, cefuroxime and nalidixic acid while CoNS isolates were sensitive to tigecycline, vancomycin, ampicillin/amoxicillin, co-amoxiclav and gentamicin. (Table 5). Most *streptococcus agalactiae* (Group B) isolates were sensitive to co-amoxiclav, cefuroxime, vancomycin, ampicillin/amoxicillin and nitrofurantoin (Table 5).

**TABLE 5:** Antimicrobial sensitivity in gram-positive bacterial isolates.

Gram-positive bacteria	*Enterococcus faecalis* (43)			Coagulase-negative staphylococci (30)			*Streptococcus agalactiae (Group B)* (15)			*Staphylococcus aureus* (8)			*Staphylococcus* spp (8)			*Enterococcus* spp (3)		
	S	R	I	S	R	I	S	R	I	S	R	I	S	R	I	S	R	I
**Ampicillin/Amoxicillin**																		
*N*	43	-	-	26	4	-	15	-	-	3	5	-	5	3	-	3	-	-
%	100	-	-	86.7	13.3	-	100	-	-	46	54	-	60	40	-	100	-	-
**Co-amoxiclav**																		
*N*	43	-	-	26	4	-	15	-	-	3	5	-	5	3	-	3	-	-
%	100	-	-	88.2	11.8	-	100	-	-	49	51	-	65	35	-	100	-	-
**Cefepime**																		
*N*	24	19	-	22	8	-	14	1	-	2	6	-	1	7	-	-	-	-
%	55	45	-	76.5	23.5	-	96	4	-	7	83	-	10	90	-	-	-	-
**Cefuroxime**																		
*N*	26	17	-	24	6	-	15	-	-	3	5	-	2	6	-	1	2	-
%	60	40	-	81.6	18.4	-	100	-	-	40	60	-	30	70	-	25	75	-
**Ciprofloxacin**																		
*N*	32	11	-	24	4	2	-	-	-	8	-	-	-	-	-	2	1	1
%	75	25	-	80	11.4	8.6	-	-	-	100	-	-	-	-	-	77.8	11.1	-
**Cotrimoxazole**																		
*N*	-	43	-	20	10	-	11	4	-	5	3	-	5	3	-	1	2	-
%	-	100	-	68.2	31.8	-	76	34	-	67	33	-	65	35	-	25	75	-
**Gentamicin**																		
*N*	-	43	-	27	3	-	6	9	-	3	5	-	5	3	-	1	2	-
%	-	100	-	91.4	8.6	-	42	58	-	46	54	-	58	42	-	4	96	-
**Nalidixic**																		
*N*	-	43	-	1	29	-	1	14	-	1	7	-	-	8	-	1	2	-
%	-	100	-	2	98	-	3	97	-	3	97	-	-	100	-	1.4	98.6	-
**Nitrofurantoin**																		
*N*	43	-	-	26	4	-	14	1	-	7	1	-	72	28	-	2	1	-
%	100	-	-	87.9	12.1	-	95	5	-	94	6	-	-	-	-	95	5	-
**Vancomycin**																		
*N*	43	-	-	-	-	-	-	-	-	-	-	-	-	-	-	3	-	-
%	100	-	-	-	-	-	-	-	-	-	-	-	-	-	-	100	-	-

S, sensitive; R, resistant; I, intermediate; *N*, numbers; %, percentages.

## Discussion

Pregnant women are at increased risk of developing UTI, mainly because of a shift in the position of the urinary tract and hormonal changes that occur throughout pregnancy, thus making it easier for bacteria to reach the kidney and leading to both symptomatic and asymptomatic bacteriuria.^2,26,27,28^

Symptomatic and asymptomatic bacteriuria are common in pregnant women. We do not perform universal screening with cultures during pregnancy. Thus, asymptomatic bacteriuria during pregnancy which is important may not be detected. This study was conducted only for symptomatic bacteriuria among pregnant women by reviewing their files.

*E. coli* was the most common gram-negative bacteria isolated in this study, which is in line with the findings of previous studies, such as that by Tandan et al. and several other studies.^1,12,13,15^ This finding suggests that most organisms causing UTI are from the lower gastrointestinal tract which acts as a reservoir for organisms such as *E. coli.*^1,9^

*Klebsiella species* was the next most common gram-negative organism isolated in this study, accounting for 14.4% of positive cultures, and is similar to the finding in a study conducted in KwaZulu-Natal (20%).^1,29^

*Enterococcus faecalis* (12.9%) was the most frequent gram-positive organism detected and had been noted as a significant bacterial isolate from women with UTI in pregnancy in other studies.^1,30^

Empiric therapy should be commenced as soon as urine samples are taken and modified once culture results become available to prevent serious morbidity.^1,31^

Hence, the recommendation of nitrofurantoin as a first line drug for the treatment of UTI in pregnancy.^7,12^ The University’s obstetric protocol recommends the use of nitrofurantoin at a dose of 100 mg orally, 6 hourly for 5 days or cefuroxime at a dose of 250 mg orally stat (this is a non-Essential Medicines List (EML) item in SA)^32^ for uncomplicated UTI and twice daily for 5 days as empirical therapy for complicated UTI.^33,34,35^

The use of nitrofurantoin in pregnancy is supported by the most recent American College of Obstetricians and Gynaecologists (ACOG) Committee opinion which concluded that in the second and third trimester it was a suitable choice. In the first trimester it can be used if there are no other suitable alternatives.^36^ The use of nitrofurantoin in pregnancy shows no increased risk for cardiovascular malformations, oral cleft, or craniosynostosis.^2,37^

Chloramphenicol, tetracyclines, and cotrimoxazole should be avoided in pregnancy.^2,34^ However, during early pregnancy chloramphenicol treatment presents little, if any, teratogenic risk to the foetus in humans.^2,38^

The exposure in utero to clindamycin, doxycycline, quinolones, and macrolides are linked to organ-specific malformations whereas exposure to amoxicillin, cephalosporins, and nitrofurantoin are not associated with major congenital malformations.^2,38^ Beta-lactams, vancomycin, nitrofurantoin, and clindamycin are generally considered safe and effective in pregnancy whereas fluoroquinolones and tetracyclines should generally be avoided in pregnancy.^2,39,40^

There was poor documentation of repeat cultures, as well as the use of prophylactic urinary antimicrobials to prevent recurrent infections, particularly in those patients with acute pyelonephritis. Only 50% of cases of pyelonephritis had repeat cultures either during admission or at follow up. There was a 0.45% incidence of pyelonephritis in this study, which is similar to reported ranges from 0.5% to 2.0% in the literature.^29^ Urinary tract infections recur in approximately 4% – 5% of pregnancies in patients with structural abnormalities of renal system and a single, postcoital dose or daily suppression with cephalexin or nitrofurantoin is an effective preventive therapy.^41^

Some studies demonstrated a relationship between UTI in pregnant women and the risk of poor perinatal outcomes.^42^ However, authors like Chen et al. concluded that there were no increased risks of adverse pregnancy outcomes in women, and neonates born to women with UTIs.^43^

### Limitations

The limitations of this study were the small sample size, missing data in the hospital file and microscopy results, symptoms at time of testing especially as symptoms of UTI and pregnancy are similar, the inherent shortcomings of a retrospective study and that it was confined to one hospital. Approximately 14% of the files were not retrieved, as some were lost. This study did not differentiate between nosocomial and community acquired infections.

Future prospective studies evaluating the impact of UTI in pregnant women in our setting using a larger sample size is recommended to address this limitation. And an improved patient record system will be beneficial to future studies.

## Conclusion

*E. coli, Klebsiella, Enterococcus faecalis* and coagulase-negative *staphylococcus* were the most common microorganisms identified in this study.^2^ Empirical therapy with oral nitrofurantoin and cefuroxime or parenteral ceftriaxone are appropriate. Most of these antibiotics are relatively safe to be used in pregnancy and breastfeeding.^2^ The choice of antimicrobial therapy in pregnancy should be determined according to the sensitivity and resistance for foeto-maternal safety.^2^
